# Tai Chi alleviates pain on fusiform-lingual and rolandic operculum-insula circuit in older participants with chronic low back pain: a randomized controlled neuroimaging trial

**DOI:** 10.3389/fnhum.2026.1739419

**Published:** 2026-05-11

**Authors:** Feng Zhang, Bo Peng, Xing Tang, Lili Peng

**Affiliations:** 1 School of Nursing, Hunan University of Chinese Medicine, Changsha, China; 2 Department of Radiology, Hospital of Chengdu University of Traditional Chinese Medicine, Chengdu, China; 3 School of Acupuncture-Moxibustion and Tuina, Chengdu University of Traditional Chinese Medicine, Chengdu, China

**Keywords:** chronic low back pain, functional connectivity, neuroplasticity, pain-related brain networks, Tai Chi

## Abstract

**Background:**

Chronic low back pain (CLBP) is a common clinical syndrome and a leading cause of disability. The high disability rate associated with CLBP is more susceptible to reduced labor capacity, poorer quality of life, and increased psychological, social, and medical burdens. Tai Chi is an effective and safe therapy for treating chronic low back pain (CLBP).

**Objective:**

This study aims to determine the effect of Tai Chi on brain function in patients with CLBP.

**Methods:**

The study included 72 participants randomly divided into a Tai Chi group and a control group. The Tai Chi group received a Yang-style 24-form Tai Chi program for 8 weeks. The control group had a waiting time of 8 weeks. Participants underwent a functional magnetic resonance imaging (fMRI) scan. The study was approved by the Ethics Committee of the Hospital of Chengdu University of Traditional Chinese Medicine in Sichuan Province (No. 2022KL-038-02) and registered with the Chinese Clinical Trial Registry under the number ChiCTR2200064977.

**Results:**

The Visual Analog Score (VAS), duration and frequency of pain, Japanese Orthopaedic Association (JOA) scores, and Oswestry Disability Index (ODI) significantly improved at 8 weeks (*p* < 0.05). Compared with the control group, the Tai Chi group showed amplitude of low-frequency fluctuation (ALFF) values significantly changed in the left fusiform and left rolandic operculum (voxel-level *p* < 0.001, cluster-level *p* < 0.05). The intensity of local spontaneous neural activity in the Tai Chi group was higher than in the control group. Tai Chi had a potential effect on the decrease of functional connectivity (FC) between the left fusiform and left lingual, between the left rolandic operculum and the right insula. (voxel-level *p* < 0.001, cluster-level *p* < 0.05). The Tai Chi group has a close correlation with brain-related regions.

**Conclusion:**

This study indicated that Tai Chi training could effectively relieve pain and improve physical function in elderly people with CLBP. The study suggested that the fusiform-lingual and rolandic operculum-insula circuits are potential treatment regions that might be the mechanism for the effect of Tai Chi.

**Clinical Trial Registration:**

https://www.chictr.org.cn, Chinese Clinical Trial Registry (ChiCTR2200064977).

## Introduction

Chronic low back pain (CLBP) is a common clinical syndrome and a leading cause of disability (Mcconnell et al., 2024). It has been reported that 21 to 68% of participants aged 60 years or older experienced CLBP in the last 12 months (de Souza et al., 2019). The increased disability rate associated with CLBP is more susceptible to weak labor capacity, reducing the quality of life, and causing psychological, social, and medical burdens (Overstreet et al., 2024). Clinical use of non-steroidal anti-inflammatory drugs, muscle relaxants, and opioids to treat CLBP is often accompanied by gastrointestinal and cardiovascular system injury and other adverse reactions (Licciardone et al., 2024). The use of opioids has increased, leading to the simultaneous growth of drug dependence and addiction, and the mortality rate has increased 4-fold (Bushey et al., 2022; Grosser et al., 2017). Clinical practice guidelines from the American College of Physicians recommend that non-pharmacologic treatment with exercise, multidisciplinary rehabilitation, acupuncture, mindfulness-based stress reduction, Tai Chi, yoga, motor control exercise, progressive relaxation, electromyography biofeedback, low-level laser therapy, operant therapy, cognitive behavioral therapy, and spinal manipulation should be used prior to the use of pharmacological treatments for patients with CLBP (Qaseem et al., 2017).

Tai Chi is a traditional Chinese mind–body practice that involves gentle, flowing, and circular movement (Chen et al., 2023). Tai Chi is an exercise of mild-to-moderate intensity that integrates physical, psychosocial, spiritual, and behavioral elements (Li F. et al., 2023). A clinical trial has shown that Tai Chi is feasible for adults over 65 years of age (Sherman et al., 2020). Several studies have demonstrated that Tai Chi can effectively alleviate pain symptoms in patients with non-specific CLBP (Zou et al., 2019). Previous systematic reviews have indicated that Tai Chi has beneficial evidence for low back pain, but high-quality trials with large sample sizes are still needed (Li Y. et al., 2023; Shi et al., 2022).

With the development of modern brain imaging technology, functional magnetic resonance imaging (fMRI) and other technologies have been used to construct brain structure and functional connectivity networks, which provide objective data to deduce central response mechanisms to how Tai Chi may operate in the brain (Gu et al., 2015; Yu et al., 2018). The physical nature of Tai Chi involves systemic bodily movement and motor control, which often interact with broader brain networks related to physical activity and hemispheric interactions (Fan et al., 2026; Yu et al., 2025). CLBP has been reported to disrupt the normal activity of the brain’s default networks even when the brain is at rest, suggesting that prolonged pain has an impact on brain structure and function. The Montreal Neurological Institute (MNI) space is a coordinate system established using a series of fMRI scans of normal human brains. The MNI template is the most commonly used in studies (Huang et al., 2025). The amplitude of low-frequency fluctuation (ALFF) is the amplitude of spontaneous neural activity intensity in the brain at rest. The ALFF meta-analysis demonstrated functional alterations in the right rolandic operculum (extending to the right insula and right inferior temporal gyrus), left inferior temporal gyrus, left middle occipital gyrus, left paracentral lobule, left postcentral gyrus, and bilateral cuneus cortex in patients with CLBP (Chen et al., 2025). Functional connections (FCs) reflect the interrelationships between different brain regions or tissues. The FCs between the right ventral dorsolateral prefrontal cortex and the right insula was significantly decreased in patients with CLBP (Zu et al., 2024). Although chronic pain is associated with reduced gray matter and impaired cognitive ability in the brain, a study by Seminowicz et al. found that effective treatment of chronic pain can restore normal brain function (Seminowicz et al., 2021).

Evidence from a neuroimaging perspective indicates that the pain-relieving effect of Tai Chi is attributed to alterations in brain activity, including the anterior cingulate cortex and insular cortex (Gu et al., 2015; Yu et al., 2018). A pilot study has suggested that moderate to large effect sizes, an important role that cortico-amygdala interactions related to Tai Chi have on pain and physical function in patients (Shen C. L. et al., 2021). In the current study, we compared the therapeutic efficacy of Tai Chi vs. a no-treatment control for the management of CLBP in elderly patients.

## Methods

### Study design

This randomized, controlled, comparative effectiveness trial recruited elderly patients with CLBP. Participants were randomly assigned to either the Tai Chi group, three times weekly for 8 weeks, or the control group, which received no intervention for 8 weeks. The study was approved by the Ethics Committee of the Hospital of Chengdu University of Traditional Chinese Medicine in Sichuan Province on June 6, 2022 (No. 2022KL-038-02), and was registered with the Chinese Clinical Trial Registry with the number ChiCTR2200064977 on October 25, 2022.

### Setting and participants

The study was conducted at the Hospital of Chengdu University of Traditional Chinese Medicine, Qing Yang District University for the Elderly, and the Ci Tang Street Community. Participants were recruited through posters, the Internet, and WeChat in Chengdu. The diagnostic criteria for CLBP followed the clinical practice guidelines of the American College of Physicians and the American Pain Society (Chou et al., 2007). Inclusion criteria were aged between 60 and 75 years. All participants had CLBP for more than 3 months. There was no infectious inflammation, fracture, or tumor in the lower back. Patients were required to sign informed consent before carrying out the study. We excluded participants who had severe joint pain or deformity, or severe spinal deformity. The patients had serious diseases known to affect mobility, such as myocardial infarction or stroke, congestive heart failure, severe chronic obstructive pulmonary disease, cancer, and other serious diseases. Exclusion criteria included participation in Tai Chi training or other clinical trials. The participants undergo blood routine and liver and kidney function examination.

### Sample size

The sample size was calculated based on the primary outcome, the visual analog score (VAS) (Chou et al., 2007). Taking a two-sided significance level of 0.05 and a power of 0.1. This study adopted a sample size of 30 participants per group. Considering a dropout rate of 20%, a total of 72 participants were included.

### Randomization

Participants were randomly assigned to two groups in a 1:1 ratio. Randomization was generated using a random number table. The random numbers were kept in sealed envelopes. Assignments were performed by an independent researcher.

### Intervention

#### Tai Chi group

Participants in the Tai Chi group were instructed in the 24-form Yang style of Tai Chi. We recruited two experienced instructors who attended a 60-min session, including a 10-min warm-up, 40-min Tai Chi training, and a 10-min cool-down. Participants were asked to attend training three times a week for 8 weeks. Attendance is recorded by the instructors. We monitored all training sessions. The participants practiced Tai Chi in group training in the community.

#### Control group

Participants in the control group received no intervention for 8 weeks. They were asked to record their pain condition. Participants reported their pain levels by telephone. Participants were restricted from other physical activities and social engagement.

### Outcome measurements

#### Primary outcome

The primary outcome was the VAS score at baseline and at 8 weeks. The VAS was used to evaluate the severity of pain in the participants (Gorji et al., 2022). The average pain was recorded using the VAS. The total score ranges from 0 to 10.

#### Secondary outcomes

The duration and frequency of pain were measured using a pain diary at baseline and 8 weeks. Participants were encouraged by the instructors to record their pain condition. The range of motion, JOA, and ODI were measured by the instructors at baseline and 8 weeks. The JOA is a 14-item questionnaire and ranges from 0 to 29, with higher scores indicating a greater function. The ODI is a 10-item questionnaire and ranges from 0 to 100, with higher scores indicating a worse impact on life. The range of motion included forward flexion, backward extension, left flexion, right flexion, left rotation, and right rotation. Stand with feet shoulder-width apart, slowly bend forward, and try to touch your toes with your fingers. Stand with both hands supporting your waist and slowly stretch your torso backward. Keep both feet together, slowly bend to the left and right sides, and slide your hands down along the outer thighs. Sit or stand, fix the pelvis, and rotate the torso left and right. The angle can be measured using a protractor. The JOA was used to evaluate low back pain. The ODI had established psychometric properties and measured pain-related disability (Berg et al., 2024).

#### Functional magnetic resonance imaging scan

Participants underwent an fMRI scan using a GE Discovery MR750 3.0 T system (General Electric, Milwaukee, WI, United States) with an 8-channel phased-array head coil at week 8. The resting-state scan duration was 15 min. The scan session included high-resolution 3-dimensional T1-weighted imaging (3D-T1W1) and blood oxygenation level-dependent functional magnetic resonance imaging (BOLD-fMRI). Participants were asked to stay awake during the scan session. The 3D-T1W1 parameters were as follows: repetition time = 1,900 ms, echo time = 2.26 ms, slice thickness = 1 mm, field-of-view = 240 × 240 mm, matrix = 256 × 256. BOLD-fMRI parameters were as follows: repetition time = 2000 ms, echo time = 30 ms, flip angle = 90°, number of slices = 32, thickness = 4.0 mm, field-of-view = 240 × 240 mm, acquisition matrix = 64 × 64 (Zhou J. et al., 2023).

### Statistical analysis

#### Clinical data analysis

The intention-to-treat method was adopted for all analyses. Two independent samples *t*-tests were used to compare VAS scores, the duration and frequency of pain, JOA, ODI, and the range of motion between the Tai Chi group and the control group. We adjusted significance levels using the Bonferroni correction. The Mann–Whitney U-test was used for non-normally distributed data. Continuous data were expressed as mean ± standard deviation. Statistical significance was set at *p* < 0.05. Missing data were processed by intention-to-treat using multiple imputation. All analyses were performed using IBM SPSS Statistics version 25 (SPSS, Inc., Chicago, IL, USA).

#### Functional magnetic resonance imaging data analysis

The fMRI data were preprocessed using Statistical Parametric Mapping 12 (SPM 12, Wellcome Department of Imaging Neuroscience, London, UK) and the Data Processing and Analysis for Brain Imaging (DPABI) software running on MATLAB (MathWorks, Inc., Natick, MA, USA). The preprocessing steps were as follows (Zhou J. et al., 2023): (1) removing the first five time points; (2) performing time layer correction, and scanning the interlayers in ascending order, starting from odd numbered layers; (3) excluding participants with head motion exceed 0.3 mm; (4) performing smoothing; (5) correction of images with T1; (6) spatial normalization through diffeomorphic anatomical registration through exponentiated lie algebra (DARTEL) (7) following the Montreal Neurological Institute (MNI) template to localize active regions of the brain (Zhou X. et al., 2023); (8) linear detrending; (9) covariate regression, including white matter, cerebrospinal fluid, and head motion signal (Friston 24); and (10) temporal filtering (0.01–0.08 Hz).

#### The amplitude of low-frequency fluctuation

The amplitude of low-frequency fluctuation (ALFF) was calculated as the average intensity of the low-frequency part of each voxel’s BOLD signal. The ALFF indicator calculation frequency range ranges from 0.01 to 0.08 Hz (Quan et al., 2022). Two independent-sample *t-*tests were used to assess differences in ALFF between participants. The threshold was set using family-wise error (FWE) correction with a voxel-level *p* < 0.001 and a cluster-level *p* < 0.05 (5,000 permutations) using DPABI and SPM 12 based on Gaussian Random Field Theory (Carotenuto et al., 2022), indicating that the difference between the two sets of data is statistically significant.

#### The resting-state functional connectivity

According to the results, ALFF was set as the voxel-wise functional connectivity (FC) analysis. First, we calculated the average time series of voxels within each region of interest (ROI). Then, we performed Pearson’s correlation calculation between the ROI average time series and the whole-brain voxel time series. Finally, a whole-brain FC map was constructed. The indicator calculation uses a standardized method to select Fisher-z and finally performs statistics on zFC. The correlation coefficients were Fisher’s transformed into Z-scores (Zhang et al., 2022). The threshold was set using FWE correction with a voxel-level *p* < 0.001 and a cluster-level *p* < 0.05, indicating that the difference between the two sets of data is statistically significant.

## Results

**Figure 1:**
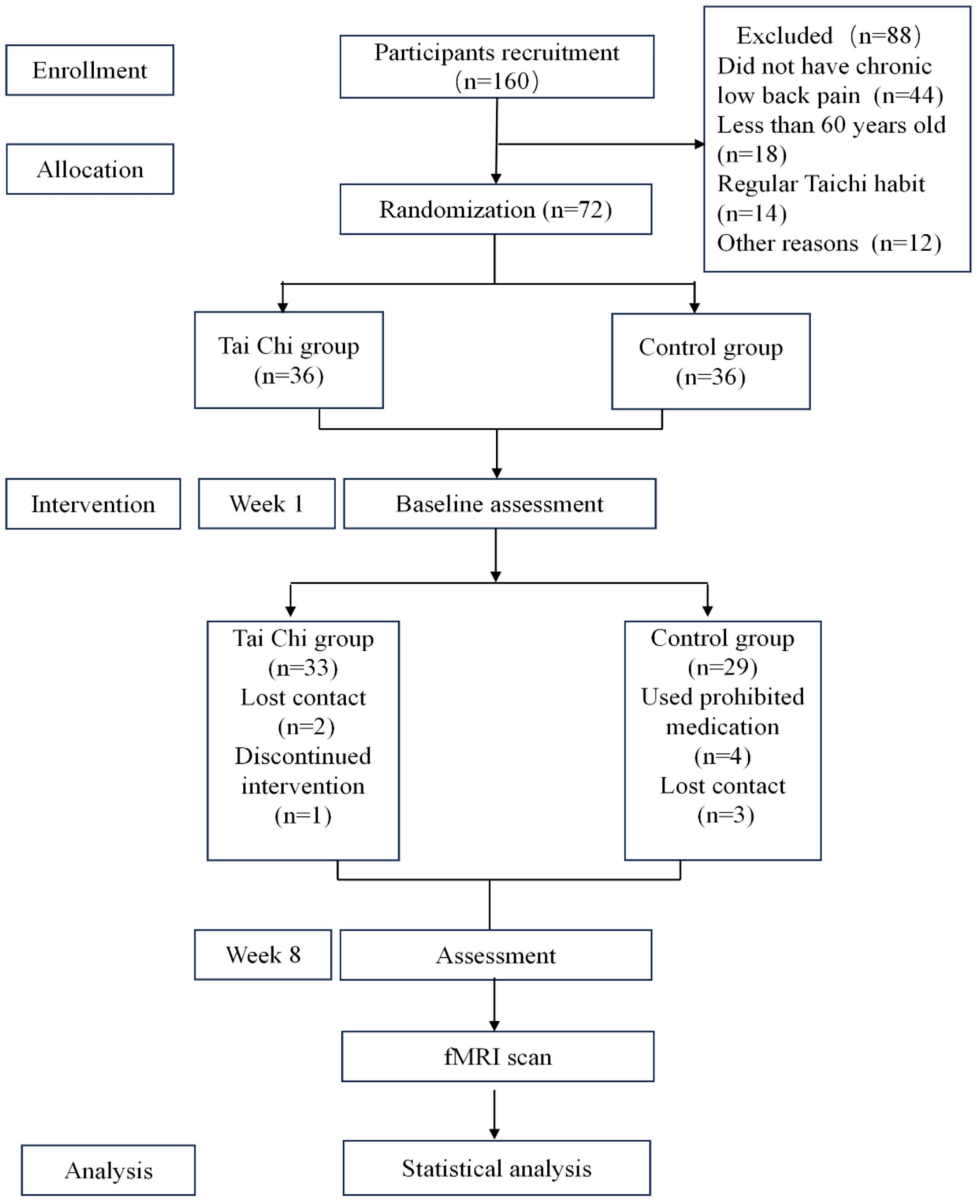
Study flow chart.

Totally, 160 patients were recruited. Figure 1 shows that 72 participants were randomly assigned to the Tai Chi or control groups.

### Baseline characteristics of participants

The characteristics of 72 eligible participants were balanced between the two groups. The mean age was 65 years, and the average duration of CLBP was 3 years. At baseline, 9.72% of participants had diabetes, and 8.33% of participants had hypertension. Approximately 5.56% of participants had other diseases (Table 1).

**Table 1:** Baseline characteristics of participants.

Characteristic	Tai Chi group (*n* = 36)	Control group (*n* = 36)
Female, *n* (%)	32 (88.89%)	28 (77.78%)
Mean age (SD), y	65.88 (4.97)	64.06 (5.87)
Pain duration, median (Q1, Q3), y	2 (0.5, 3.75)	2 (1, 3.5)
Participants with disease, *n* (%)		
Coronary heart disease	0 (0)	0 (0)
Diabetes	4 (11.11)	3 (8.33)
Hypertension	3 (8.33)	3 (8.33)
Other	2 (5.56)	2 (5.56)

### Primary outcome

The primary outcomes are summarized in Table 2. The mean VAS score was 4.21 ± 2.04 in the Tai Chi group and 4.39 ± 1.56 in the control group. After 8 weeks, compared with the control group, VAS scores significantly improved for participants in the Tai Chi group at week 8 (95%CI, 0.11–2.5; *p* = 0.03) (Table 2).

**Table 2:** Primary and secondary outcomes.

Outcome	Tai Chi group (*n* = 32)	Control group (*n* = 30)	Tai Chi group vs. control group	
			Adjusted mean difference (95%CI)	*p* value
Primary outcome				
Mean VAS (SD)				
Baseline	4.21 (2.04)	4.39 (1.56)	(−1.28 to 1.62)	0.81
Week 8	1.79 (1.42)	3.09 (1.45)	(0.11 to 2.5)	0.03
Secondary outcomes				
Mean duration of pain (SD)				
Baseline	2.16 (1.04)	2.26 (1.54)	(−1.07 to 1.27)	0.86
Week 8	0.85 (0.71)	2.27 (1.45)	(0.43 to 2.41)	0.01
Mean frequency of pain (SD)				
Baseline	2.97 (1.73)	2.51 (1.71)	(−2.24 to 1.32)	0.59
Week 8	0.96 (0.79)	2.13 (1.25)	(0.1 to 2.24)	0.03
Mean range of motion (SD)				
FF				
Baseline	65.78° (20.76°)	63.63° (20.44°)	(−20.56 to 16.26)	0.81
Week 8	69.99° (15.96°)	75.5° (12.8°)	(−7.63 to 18.69)	0.39
BE				
Baseline	17.42° (4.48°)	21.51° (9.02°)	(−2.09 to 10.25)	0.18
Week 8	19.24° (6.09°)	18.02° (6.21°)	(−6.67 to 4.24)	0.65
LF				
Baseline	21.42° (6.57°)	23.3° (5.93°)	(−3.75 to 7.5)	0.49
Week 8	22.63° (5°)	25.46° (5.02°)	(−1.59 to 7.26)	0.2
RF				
Baseline	18.43° (6.41°)	23.86° (8.92°)	(−1.4 to 12.25)	0.11
Week 8	20.35° (7°)	19.44° (5.64°)	(−6.67 to 4.87)	0.75
LR				
Baseline	43.83° (21.67°)	44.45° (12.09°)	(−15.46 to 16.7)	0.94
Week 8	39.46° (13.05°)	39.93° (8.08°)	(−9.67 to 10.62)	0.92
RR				
Baseline	45.02° (17.47°)	46.2° (10.34°)	(−11.94 to 14.31)	0.85
Week 8	44.59° (12.4°)	50.92° (9.67°)	(−3.83 to 16.5)	0.21
JOA				
Baseline	21.7 (3.34)	22.36 (3.08)	(−2.26 to 3.59)	0.64
Week 8	25.09 (2.12)	22.25 (2.55)	(−5.1 to −0.58)	0.02
ODI				
Baseline	9 (4.78)	10.79 (5.74)	(−2.23 to 5.8)	0.37
Week 8	4.38 (2.6)	8 (5.04)	(0.58 to 6.67)	0.02

SD, standard deviation; VAS, visual analogue score; FF, forward flexion; BE, backward extension; LF, left flexion; RF, right flexion; LR, left rotation; RR, right rotation; JOA, Japanese Orthopaedic Association Scores; ODI, Oswestry Disability Index.

### Secondary outcomes

After 8 weeks of treatment, patients in the Tai Chi group showed significantly greater improvement in the duration of pain compared to the control group (0.85 ± 0.71 vs. 2.27 ± 1.45; *p* < 0.05). Compared with the control group, the Tai Chi group also showed a significant reduction in the frequency of pain at week 8 (0.96 ± 0.79 vs. 2.13 ± 1.25; *p* < 0.05). However, no statistically significant differences were observed in the range of motion (*p >* 0.05). The JOA scores in the Tai Chi group increased from 21.7 ± 3.34 at baseline to 4.38 ± 2.6 after treatment, compared with a smaller reduction from 22.36 ± 3.08 to 22.25 ± 2.55 in the control group. At 8 weeks, the Tai Chi group continued to show a significantly larger improvement in ODI scores compared to the control group (4.38 ± 2.6 vs. 8 ± 5.04; *p* < 0.05) (Table 2).

### Adverse events and intervention attendance rates

Throughout the study period, we monitored participants for adverse events. No adverse events were found in the study. A total of 72 participants were included in the compliance evaluation, and 62 participants had a compliance rate of 86.11%.

### The amplitude of low-frequency fluctuation in participants

**Figure 2:**
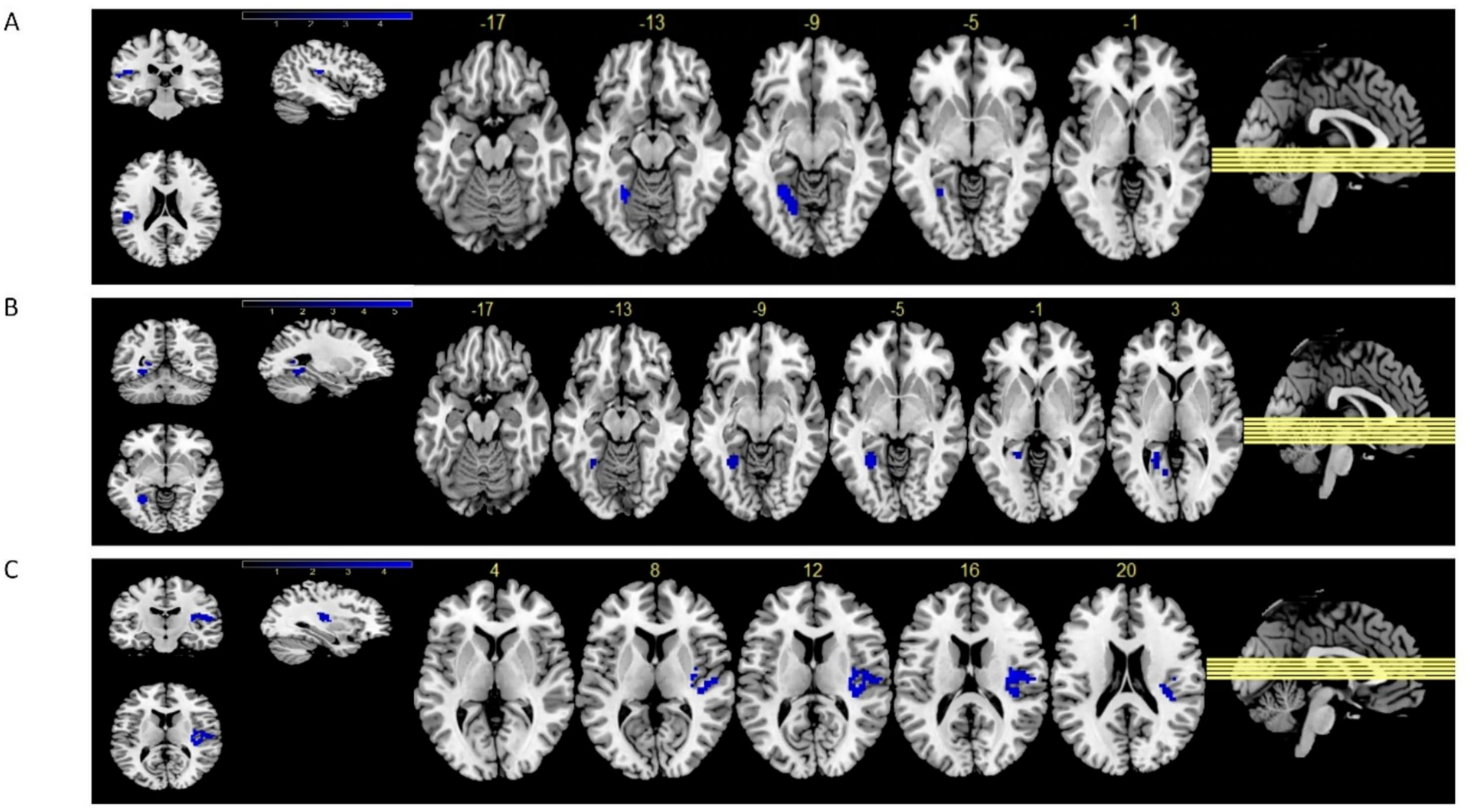
Comparison of the ALFF and FC between the Tai Chi and Control groups. Compared with the Control group, ALFF values decreased in the left fusiform and left rolandic operculum for participants in Tai Chi group (voxel-level *p* < 0.001, cluster-level *p* < 0.05) **(A)**. There were significantly decreased FC between the left fusiform and left lingual (voxel-level *p* < 0.001, cluster-level *p* < 0.05) **(B)**. Compared with the Control group, participants had decreased FC between the left rolandic operculum and right insula in Tai Chi group (voxel-level *p* < 0.001, cluster-level *p* < 0.05) **(C)**.

As shown in Figure 2A, compared with the Control group, ALFF values decreased in the left fusiform and the left rolandic operculum for participants in the Tai Chi group (voxel-level *p* < 0.001, cluster-level *p* < 0.05).

### Resting-state functional connectivity analysis

The ALFF values with significant differences in the left fusiform and left rolandic operculum were taken as ROIs. The results demonstrated that there was a significant decrease in FC between the left fusiform and the left lingual (voxel-level *p* < 0.001, cluster-level *p* < 0.05, Figure 2B; Table 3). Compared with the control group, participants in the Tai Chi group had a decreased FC between the left rolandic operculum and right insula(voxel-level *p <* 0.001, cluster-level *p <* 0.05, Figure 2C; Table 3).

**Table 3:** Comparisons of the brain changes between the Tai Chi and control groups.

Contrast	Cluster	Brain region	T	MNI coordinates		
				X	Y	Z
Tai Chi < Control (ALFF)	46	Fusiform_L	4.7492	−27	−54	−9
	43	Rolandic_Oper_L	4.8467	−45	−27	21
Tai Chi < Control (FC)	91	Lingual_L	5.5049	−27	−54	−6
	135	Insula_R	4.7301	33	−15	12

ALFF, amplitude of low frequency fluctuation; FC, functional connectivity; MNI, Montreal Neurological Institute; Rolandic_Oper, rolandic operculum; Temporal_Mid, middle temporal; L, left; R, right; voxel-level *p* < 0.001, cluster-level *p* < 0.05.

## Discussion

In this neuroimaging study, we explored the modulating effects of Tai Chi on brain activity in elderly patients with CLBP. We found that 8-week Tai Chi training in the elderly participants with CLBP could decrease pain intensity, suggesting that the Tai Chi group had a significantly greater reduction in pain than those who received no training. Furthermore, Tai Chi led to significant improvements in quality of life. No adverse events were reported from the participants. However, a previous systematic review and meta-analysis found the efficacy of Tai Chi for CLBP uncertain (Wen et al., 2022). The meta-analysis showed that the quality of clinical trials is low, and higher-quality research needs to be included. The neuroimaging results demonstrated that Tai Chi could significantly elicit activity in the fusiform-lingual and rolandic operculum-insula circuits in elderly patients with CLBP. These neural circuits are closely related to pain perception.

Previous studies have demonstrated that Tai Chi effectively reduces VAS scores in the treatment of non-specific CLBP (Zou et al., 2019). These findings suggest that Tai Chi provides greater symptom improvement of pain for at least 8 weeks compared to no training. A previous study found that Tai Chi provided several functional, psychospiritual, and social support benefits for older adults with CLBP (Lee et al., 2020). Future research focuses on the relationship of Tai Chi on improving both chronic low back pain and mood. Previous studies on the effectiveness of Tai Chi in improving CLBP were mostly based on subjective scale evaluations, and objectives lacked quantitative indicators (Petri et al., 2026; Sotiropoulos et al., 2025). A strength of this trial is to assess and quantify pain. The way the participants wrote the pain diary, recording the time and frequency of pain occurrence, is more consistent with the clinical management of pain improvement. In our study, there was no difference in lumbar spine mobility. Lumbar spine mobility is related to height and weight. The results of our study show that Tai Chi can reduce functional impairment and improve the quality of life. A network meta-analysis has also confirmed that Tai Chi can improve quality of life (Xia et al., 2025). No Tai Chi training-related adverse events were reported in our study.

Comparison of ALFF and FC between the Tai Chi and control groups (voxel-level *p <* 0.001, cluster-level *p <* 0.05). Compared with the control group, ALFF values decreased in the left fusiform gyrus and left rolandic operculum for participants in the Tai Chi group (Figure 2A). There was significantly decreased FC between the left fusiform gyrus and left lingual in the Tai Chi group (Figure 2B). Participants in the Tai Chi group had decreased FC between the left rolandic operculum and the right insula (Figure 2C).

This study found that Tai Chi can significantly decrease the intensity of brain activity in the left fusiform gyrus and the left rolandic operculum. It has been reported that a significant decrease in ALFF was observed in the fusiform gyrus in patients with discogenic low-back and leg pain (Zhou et al., 2018). Another study showed that Tai Chi significantly affected inhibitory control performance and spontaneous neural activity, which were associated with significantly increased fALFF in the left medial superior frontal gyrus and the right fusiform gyrus, and decreased fALFF in the right dorsolateral superior frontal gyrus and the right paracentral lobule (Shen Q. Q. et al., 2021). Inhibition is a cognitive control process that allows individuals to suppress dominant and automatic responses to goal-irrelevant stimuli when needed. It plays significant and intricate roles in different dimensions of thinking and behavioral processes, including attention, emotional perception, and emotional regulation. These findings suggest that the fusiform gyrus is not only associated with low back pain but also with cognitive function. The study results demonstrate that brain areas, including the paracingulate gyrus, insula/secondary somatosensory area, inferior frontal gyrus, temporal gyrus, and fusiform gyrus, predict the development of CLBP (Baran et al., 2022). The right lingual gyrus is important for the processing of pain (Wen et al., 2022). Rolandic operculum and temporoparietal junction have been identified as common potential targets for chronic pain (Kong et al., 2024). Furthermore, pain-related activation in key brain regions, including the contralateral posterior insula, bilateral ventral anterior insula, ventral anterior cingulate, dorsolateral prefrontal cortex, and nucleus accumbens, may serve as biomarkers for mind–body interventions in CLBP (Strigo et al., 2024). With ongoing advances in neuropathological research, changes in brain structural neuroplasticity in patients with CLBP have been revealed. Neuroplasticity is an important mechanism for self-regulation in the brain. Future research on CLBP will focus on structural neuroplasticity (Kuang et al., 2023; Yu et al., 2023; Zou and Hao, 2024). However, our findings demonstrate that Tai Chi can regulate the fusiform-lingual and rolandic operculum-insula circuits in elderly patients with CLBP. Our results provide a valuable reference for the clinical application of Tai Chi in the treatment of CLBP.

This study has several limitations. First, given the small sample size for a neuroimaging trial, there is a high potential risk of Type II errors in the fMRI analyses. Second, the study evaluated outcomes only at the end of the 8-week intervention without a follow-up period. Future research will increase the design of follow-up periods. Third, this study lacks control of healthy participants. Fourth, the results may be influenced by age, gender, and inter-individual variability. Finally, we did not perform fMRI scans at baseline, nor did we measure potential mediator variables. Future multicenter trials with larger sample sizes are warranted to assess the long-term effects of Tai Chi and to further investigate the potential mechanism of the brain network.

## Conclusion

This study indicates that an 8-week Tai Chi training intervention can effectively relieve pain and improve physical function in older participants with CLBP. This study indicated that Tai Chi training could effectively relieve pain and improve physical function in older participants with CLBP. Furthermore, the study suggests that the fusiform-lingual and rolandic operculum-insula circuits are potential treatment regions that might be a mechanism for the effect of Tai Chi. These findings provided functional brain biomarkers for future mind–body interventions in older participants with CLBP.

## Data Availability

The raw data supporting the conclusions of this article will be made available by the authors, without undue reservation.
